# Early Recognition of Hypermagnesemia-Induced Prolonged Muscle Relaxation and Delayed Arousal Through Ionized Magnesium Measurement

**DOI:** 10.7759/cureus.79865

**Published:** 2025-03-01

**Authors:** Akira Okada, Takao Kato, Kunihide Okubo, Yuki Kurokawa, Kaoru Koyama

**Affiliations:** 1 Department of Anesthesiology, Saitama Medical Center, Saitama Medical University, Kawagoe, JPN

**Keywords:** acute kidney injury, hypermagnesemia, ionized magnesium, magnesium oxide, prolonged muscle relaxation

## Abstract

Hypermagnesemia is often iatrogenic and special attention is required in patients taking magnesium oxide, particularly those with impaired renal function or gastrointestinal obstruction. We report a case of a 60-year-old man who developed obstructive ileus due to sigmoid colon cancer. He had been prescribed magnesium oxide (2000 mg/day) and magnesium citrate (34 g) preoperatively. Postoperatively, he exhibited prolonged muscle relaxation and delayed awakening. Blood gas analysis revealed a significantly elevated ionized magnesium level of 3.53 mmol/L (reference range: 0.45-0.67 mmol/L). Continuous hemodiafiltration was promptly initiated, leading to patient awakening on postoperative day one and transfer to the general ward on postoperative day three. Retrospective analysis confirmed a total serum magnesium level of 14.1 mg/dL (reference range: 1.8-2.4 mg/dL) immediately after surgery.

In this case, magnesium oxide accumulation due to obstructive ileus, combined with renal impairment caused by septic shock, likely contributed to the development of hypermagnesemia. The use of a blood gas analyzer capable of measuring ionized magnesium allowed for early recognition and early therapeutic intervention. This case highlights the risk of hypermagnesemia even with short-term magnesium oxide use in patients with gastrointestinal obstruction or renal dysfunction. Furthermore, it underscores the importance of magnesium monitoring in critically ill patients and the utility of ionized magnesium measurement in clinical practice.

## Introduction

Hypermagnesemia is primarily caused by excessive magnesium intake, renal failure, or abnormal magnesium distribution, with most cases being iatrogenic [1]. Magnesium oxide is commonly prescribed for constipation and other gastrointestinal disorders. However, recent reports have documented cases of severe hypermagnesemia leading to life-threatening symptoms, even in patients with normal renal function or those receiving doses lower than the standard therapeutic range [2,3]. The Pharmaceuticals and Medical Devices Agency (PMDA) issued three warnings in 2008, 2015, and 2020, emphasizing the need for caution, particularly in patients undergoing long-term treatment, those with renal impairment, elderly patients, and those with constipation [4-6].

In such cases, it is crucial to suspect hypermagnesemia based on symptoms and findings, and to monitor magnesium levels. While total magnesium (tMg) is commonly measured in clinical practice, ionized magnesium (iMg) is the physiologically active form in the body [7]. The measurement of tMg is typically performed through biochemical testing, which requires approximately 30 minutes to 1 hour, and in some institutions, it is outsourced, leading to delays of several days. In contrast, iMg can be measured rapidly within minutes using specific blood gas analyzers. However, the availability of such analyzers remains limited. In situations where rapid magnesium measurement is not feasible, hypermagnesemia is often diagnosed only after severe symptoms, such as coma or cardiac arrest, already manifest [2].

Here, we report a case of hypermagnesemia that was promptly diagnosed using blood gas analysis for iMg, allowing for early therapeutic intervention. This case highlights the importance of timely magnesium monitoring and the clinical utility of iMg measurement in critical settings.

## Case presentation

A 60-year-old man (height: 170 cm, weight: 60 kg) with a history of cholelithiasis presented with abdominal distension and loss of appetite for two weeks. Further examination led to a diagnosis of sigmoid colon cancer and surgery was planned. Although anemia was noted, renal function was initially normal. To manage bowel movements, oral magnesium oxide (2000 mg/day) was initiated six days before surgery. Two days before surgery, he was prescribed magnesium citrate (34 g) and was temporarily discharged. The number of bowel movements was one to five times a day until discharge. However, his symptoms worsened the following day, leading to emergency readmission. On admission, his blood pressure was 92/68 mmHg, heart rate was 95 bpm, and he was drowsy and had abdominal pain, but electrocardiogram abnormalities were observed. Laboratory findings indicated renal dysfunction, liver dysfunction, and thrombocytopenia, raising suspicion of sepsis and tumor-induced obstructive ileus. Consequently, laparoscopic colostomy was performed (Table 1).

**Table 1:** Blood test results on the day of surgery.

Variables	Test values	Reference ranges	Units
Albumin	1.9	4.1-5.1	g/dL
Aspartate transaminase	210	13-30	U/L
Alanine transaminase	95	7-23	U/L
Lactate dehydrogenase	1049	124-222	U/L
Creatinine	1.05	0.46-0.79	mg/dL
Blood urea nitrogen	27	8-20	mg/dL
Sodium	130	138-145	mEq/L
Potassium	4.0	3.6-4.8	mEq/L
Chloride	101	101-108	mEq/L
Total bilirubin	1.0	1.4-1.5	mg/dL
C-reactive protein	3.45	0-0.14	mg/dL
Activated partial thromboplastin time	28.8	24.1-31.7	s
Prothrombin time (international normalized ratio)	1.55	0.8-1.2	-
Fibrinogen	294	193-412	mg/dL
White blood cell	11.2	3.3-8.6	10^3^/µL
Red blood cell	3.89	3.86-4.92	10^6^/µL
Hemoglobin	8.2	11.6-14.8	g/dL
Platelet count	44	158-348	10​​​​​​​^3^/µL

Anesthesia was induced with propofol, rocuronium 50 mg (0.8 mg/kg), and fentanyl, and maintained with desflurane, remifentanil, and fentanyl. No additional rocuronium was administered during surgery, but train-of-four (TOF) count remained at 0 throughout the procedure. The surgery was uneventful, lasting 49 minutes, with a total fluid administration of 1700 mL, minimal blood loss, and no urine output. Intraoperative findings confirmed obstructive ileus without evidence of gastrointestinal perforation. Postoperatively, 200 mg of sugammadex was administered; however, the patient failed to regain consciousness and exhibited prolonged neuromuscular blockade (TOF count: 0). Additional doses of naloxone (0.2 mg) and sugammadex (200 mg) were given, but no improvement was observed, necessitating continued intubation and ICU admission.

**Figure 1:**
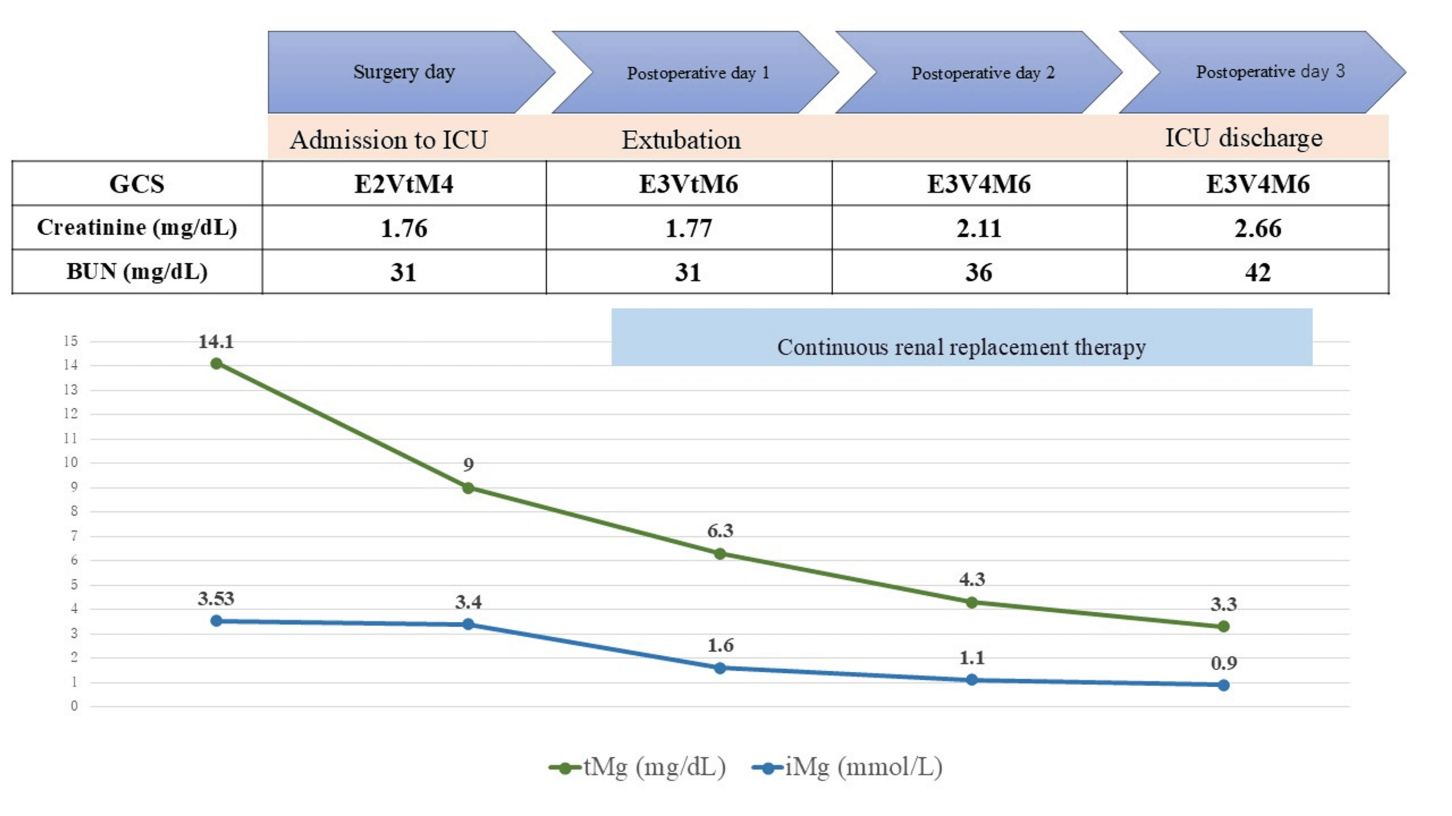
Postoperative course of the patient. tMg reference range: 1.8-2.5 mg/dL, iMg reference range: 0.45-0.67 mmol/L, creatinine reference range: 0.46-0.79 mg/dL, BUN reference range: 8-20 mg/dL tMg: total serum magnesium concentration; iMg: ionized magnesium concentration; GCS: Glasgow Coma Scale; BUN: blood urea nitrogen

Postoperative blood gas analysis revealed severe hypermagnesemia, with an iMg level of 3.53 mmol/L (reference range: 0.45-0.67 mmol/L) (Table 2). Upon ICU admission, the patient was hemodynamically stable with norepinephrine and dobutamine infusion but had a Sequential Organ Failure Assessment (SOFA) score of 9. Given the presence of septic shock and worsening renal function, continuous hemodiafiltration was initiated. iMg levels rapidly declined, leading to an improvement in consciousness, extubation on postoperative day one, and transfer to the general ward on postoperative day three (Figure 1).

**Table 2:** Blood gas analysis immediately after surgery. Ventilator settings were as follows - FiO₂: 0.6, tidal volume: 500 mL, positive end-expiratory pressure: 5 cmH₂O, and respiratory rate: 12 breaths/minute.

Variables	Test values	Reference ranges	Units
Potential hydrogen	7.284	7.35-7.45	-
Arterial carbon dioxide pressure	50.2	35.0-48.0	mmHg
Arterial oxygen pressure	203.6	>80	mmol/L
Ionized sodium	131	135-148	mmol/L
Ionized potassium	3.92	3.5-4.5	mmol/L
Ionized chloride	102.7	98-107	mmol/L
Ionized calcium	1.33	1.12-1.32	mmol/L
Ionized magnesium	3.53	0.45-0.67	mmol/L
Lactate	5.6	0.5-1.6	mmol/L

Subsequent biochemical analysis of retained blood samples confirmed a tMg level of 14.1 mg/dL (reference range: 1.8-2.4 mg/dL) immediately postsurgery. The patient was successfully weaned off dialysis by postoperative day 31 and was discharged home on postoperative day 48.

## Discussion

We encountered a case of prolonged neuromuscular blockade due to hypermagnesemia in a patient taking magnesium oxide who developed obstructive ileus and subsequent postoperative renal dysfunction. In this case, oral magnesium oxide was initiated six days before surgery, and magnesium citrate was prescribed two days preoperatively. Despite the short duration of magnesium intake, prolonged retention of magnesium oxide in the intestines due to obstructive ileus likely contributed to the development of hypermagnesemia [3]. Additionally, the patient experienced septic shock due to disruption of the gastrointestinal mucosal barrier caused by obstructive ileus, leading to renal impairment, which further exacerbated hypermagnesemia.

Delayed awakening after surgery has multiple differential diagnoses, including acidosis, hypoglycemia, infection, and cerebrovascular events [8]. There have been reports of hypermagnesemia causing impaired consciousness [9]. At our institution, blood gas analyzers capable of measuring iMg (Stat Profile pHOx Ultra; Waltham, MA: Nova Biomedical) are routinely available in both the operating room and intensive care unit, allowing for early detection and timely intervention.

In this case, the postoperative tMg level was markedly elevated at 14.1 mg/dL, which could have led to fatal complications such as cardiac arrest or complete atrioventricular block, in addition to neuromuscular blockade and delayed awakening [10]. However, the early recognition of hypermagnesemia via iMg measurement enabled the prompt initiation of continuous hemodiafiltration in the intensive care unit, preventing further complications. Previous studies have reported that in critically ill patients, iMg and tMg levels do not always correlate, and relying solely on tMg measurements may lead to over or underestimation of magnesium abnormalities [11-13]. Future studies should investigate the optimal reference range for magnesium levels using iMg as a more reliable marker.

In this case, hypermagnesemia was detected after surgery. However, the patient was taking oral magnesium oxide, and there was also the possibility of deterioration of renal function. In addition, the patient had hypotension and somnolence before entering the operating room, but this was judged to be due to sepsis caused by gastrointestinal perforation rather than hypermagnesemia. During the operation, blood gas analysis was not performed because the operation time was short and the amount of bleeding was small. Suppose a blood gas analysis was performed before or after admission to the operating room to investigate hypermagnesemia. In this case, earlier diagnosis and treatment might have been possible.

As demonstrated in this case, even a short period of oral magnesium oxide administration can lead to unexpected hypermagnesemia in patients with ileus and associated renal dysfunction. Furthermore, while hypermagnesemia is a concern, hypomagnesemia is actually more prevalent among critically ill patients [14]. Given the importance of maintaining magnesium homeostasis, routine monitoring of magnesium levels in critically ill patients is essential. The use of blood gas analyzers capable of iMg measurement should be considered a valuable tool for early diagnosis and management of magnesium imbalances in critical care settings.

## Conclusions

In this case, the use of a blood gas analyzer capable of measuring ionized magnesium allowed for the early recognition and intervention of unexpected hypermagnesemia, which was the underlying cause of delayed awakening. Routine iMg measurement should be considered a standard clinical practice for critically ill patients to facilitate timely and accurate assessment of magnesium abnormalities.
